# Volatile Compound Profiling and Quality Assessment of Sweet Fermented High-Amylose Rice: A Comparative GC-MS Analysis with Traditional Glutinous Rice Fermentation

**DOI:** 10.3390/molecules31060937

**Published:** 2026-03-11

**Authors:** Kamonwan Chucheep, Nongnuch Siriwong, Zee Wei Lai, Naree Phanchindawan

**Affiliations:** 1 Department of General Science and Liberal Arts, King Mongkut’s Institute of Technology Ladkrabang, Prince of Chumphon Campus, Chumphon 86160, Thailand; 2 Department of Home Economics, Faculty of Agriculture, Kasetsart University, Bangkhen Campus, Bangkok 10900, Thailand; 3 School of Biosciences, Faculty of Health and Medical Sciences, Taylor’s University, Subang Jaya 47500, Selangor, Malaysia; zeewei.lai@taylors.edu.my; 4 Centre for Active Living, Taylor’s University, Subang Jaya 47500, Selangor, Malaysia; 5 Scientific Laboratory Service Center, King Mongkut’s Institute of Technology Ladkrabang, Prince of Chumphon Campus, Chumphon 86160, Thailand; naree.ph@kmitl.ac.th

**Keywords:** Lueang Patew Chumphon rice, sweet fermented rice, volatile compounds, GC-MS analysis, traditional fermentation, degree of gelatinization, mineral bioavailability

## Abstract

High-amylose Lueang Patew Chumphon (LPC) rice, a Thai geographical indication variety, represents an underutilized resource for functional food development. This study investigated sweet fermented LPC rice (SFLPC) compared to conventional sweet fermented glutinous rice (SFGR) through comprehensive microbial, chemical, and nutritional characterization. Starter cakes contained *Aspergillus* sp., *Rhizopus stolonifer*, and *Pediococcus pentosaceus* (>99% similarity by ITS/16S rRNA sequencing and MALDI Biotyper). Both varieties demonstrated comparable fermentation with pH reductions to ~3.5 and lactic acid production (~6 g/L). GC-MS analysis with mass spectral library matching and Linear Retention Index (LRI) comparison tentatively annotated twelve volatile compounds. Absolute peak area analysis revealed distinct variety-specific profiles: SFGR was characterized by significantly higher ethyl palmitate (75.89 ± 19.30 vs. 16.80 ± 7.21 × 10^6^, *p* = 0.008) and isobutyl alcohol (33.09 ± 3.56 vs. 23.53 ± 1.71 × 10^6^, *p* = 0.014), exclusive ethyl dodecanoate (44.87 ± 20.60 × 10^6^), and exclusive 2,4-di-tert-butylphenol, while SFLPC showed exclusive ethyl acetate formation. Isoamyl alcohol was the dominant volatile in both varieties, with comparable absolute peak areas (273.91 ± 22.65 vs. 267.54 ± 28.78 × 10^6^, ns). SFLPC demonstrated superior mineral retention (2.1-fold phosphorus, 1.9-fold potassium and magnesium) and enhanced antioxidant capacity (IC_50_: 3.30 vs. 5.20 μg/mL, representing 36% improvement). Degree of gelatinization analysis validated comparable starch gelatinization (32.5–40.1%) despite different cooking methods, confirming volatile differences arose from rice variety rather than processing. These findings demonstrate high-amylose LPC rice as a promising fermented food substrate offering enhanced nutritional properties and volatile compound profiles through traditional fermentation.

## 1. Introduction

Rice serves as the primary staple food across Asian countries, particularly in Southeast Asia, where Thailand ranks among the top ten rice-consuming nations globally and is one of the world’s leading rice exporters [1]. Thai rice varieties are traditionally classified into four main categories: Thai Hom Mali rice (Jasmine rice), glutinous rice, white rice, and unpolished rice. Among these, Lueang Patew Chumphon (LPC) rice belongs to the white rice category and represents a distinctive traditional variety indigenous to Pathew District, Chumphon Province, southern Thailand, thriving in lowland areas with acidic and brackish saline soils [2]. The variety received official geographical indication (GI) recognition from the Department of Intellectual Property Thailand on 30 December 2008 [3]. LPC rice is characterized by exceptionally high amylose content (>25%), which confers several functional advantages, including enhanced resistant starch formation, superior mineral bioavailability, improved protein digestibility, slower glucose release, and a lower glycemic index compared to conventional rice varieties [4,5,6]. These properties make LPC rice a promising candidate for functional food development [7].

Traditional Thai sweet fermented rice (Khao Mak) is a complex fermented food system involving multiple microorganisms—including yeasts, lactic acid bacteria, and fungi—that collectively drive starch hydrolysis, ethanol production, and organic acid accumulation, contributing to distinctive flavor profiles, nutritional enhancement, and preservation characteristics [8,9]. The application of molecular identification techniques, including ITS/16S rRNA sequencing, has become standard practice for characterizing fermentative microorganisms in such systems [9]. The fermentation process significantly alters the volatile compound composition, creating unique organoleptic properties that determine consumer acceptance and product quality [10]. Gas Chromatography-Mass Spectrometry (GC-MS) has emerged as the standard tool for volatile compound profiling in fermented foods, enabling systematic characterization of headspace volatile constituents [11]. It should be noted that determination of aroma-active compounds specifically requires GC-Olfactometry (GC-O), which was beyond the scope of the present study.

Despite the well-documented health advantages of high-amylose rice, LPC rice remains underutilized due to consumer preference for softer-textured, low-amylose varieties. Limited research exists on the comprehensive quality assessment of high-amylose rice varieties in fermented applications, particularly regarding volatile compound formation, nutritional enhancement, and mineral bioavailability compared to conventional fermented rice products [5]. Therefore, the specific objectives of this study were to: (a) characterize and compare volatile compound profiles of sweet fermented LPC rice (SFLPC) and sweet fermented glutinous rice (SFGR) using HS-SPME-GC-MS analysis; (b) evaluate compositional and nutritional parameters between the two fermented products; and (c) provide scientific evidence for the potential of high-amylose rice varieties in innovative fermented food development.

## 2. Results

### 2.1. Microbial Community and Fermentation Dynamics 

DNA sequencing and BLAST analysis identified three key microbial species from traditional starter cakes: *Aspergillus* sp. (100% similarity), *Rhizopus stolonifer* (99.84% similarity), and *Pediococcus pentosaceus* (99.80% similarity). These microorganisms established the foundation for subsequent fermentation processes.

At fermentation initiation (day 0), both fungal species (*Aspergillus* sp. and *R. stolonifer*) were present alongside *P. pentosaceus*, facilitating initial starch hydrolysis through amylase enzyme production. By day 1, fungal populations were no longer detectable, while *Candida tropicalis* emerged as the dominant yeast species alongside *P. pentosaceus*. This microbial succession pattern reflected the changing environmental conditions during fermentation, particularly decreasing pH and oxygen availability.

Both rice varieties demonstrated comparable fermentation patterns over 48 h (Table 1). pH reduction showed large effect sizes in both varieties: SFLPC decreased 43% from 6.15 ± 0.02 to 3.50 ± 0.01 (Cohen’s d = 2.1, *p* < 0.001) while SFGR decreased 39% from 5.78 ± 0.11 to 3.55 ± 0.00 (Cohen’s d = 1.9, *p* < 0.001). The initial pH difference between varieties (0.37 units) represented a moderate effect size (Cohen’s d = 0.6), indicating statistically significant but practically moderate differences in initial fermentation conditions.

Total soluble solids (TSS) increases demonstrated large effect sizes, with SFGR showing a 56% higher final concentration (33.47 ± 0.12 °Brix) compared to SFLPC (21.47 ± 0.06 °Brix), representing a difference of 12.0 °Brix with large practical significance. SFGR exhibited a 4.3-fold increase while SFLPC showed a 6.4-fold increase from baseline values (Table 1).

Ethanol production showed small effect sizes between varieties, with concentrations differing by only 3% (3.60 ± 0.11% *v*/*v* in SFLPC vs. 3.72 ± 0.12% *v*/*v* in SFGR). Lactic acid concentrations increased 2.4-fold in both varieties, reaching over 6000 mg/L with negligible between-variety differences (0.2% difference, representing minimal practical significance). The concurrent production established dual preservation mechanisms through low pH and elevated organic acid concentrations.

### 2.2. Volatile Compounds Profiles

Twelve volatile compounds were tentatively annotated using mass spectral library matching (match factor > 90%) and Linear Retention Index (LRI) comparison, comprising four alcohols, five esters, one aldehyde, one ketone, and one phenolic compound (Table 2). Eight compounds were confirmed at MSI Level 1 using authentic commercial reference standards; four compounds were retained as MSI Level 2 tentative annotations. Results are expressed as mean absolute peak area (×10^6^ ± SD, *n* = 3), in accordance with HS-SPME best practice for comparative profiling analyses [12,13].

Isoamyl alcohol was the dominant compound in both varieties, formed via the Ehrlich pathway from leucine catabolism [10]. Absolute peak area analysis showed comparable levels in SFLPC (273.91 ± 22.65 × 10^6^) and SFGR (267.54 ± 28.78 × 10^6^), with no statistically significant between-variety difference (*p* = 0.778, ns). Isobutyl alcohol was significantly higher in SFGR (33.09 ± 3.56 × 10^6^) than SFLPC (23.53 ± 1.71 × 10^6^, *p* = 0.014), consistent with greater branched-chain amino acid catabolism in the glutinous rice fermentation system. Acetaldehyde (SFLPC: 6.98 ± 0.88; SFGR: 8.24 ± 1.14 × 10^6^, ns), menthol (SFLPC: 16.55 ± 8.57; SFGR: 24.80 ± 7.57 × 10^6^, ns), and acetoin (SFLPC: 20.75 ± 7.51; SFGR: 21.26 ± 1.06 × 10^6^, ns) showed no significant between-variety differences. 2-Ethyl-1-hexanol was detected exclusively in SFGR (17.09 ± 2.51 × 10^6^, *n* = 2), while ethyl acetate was detected exclusively in SFLPC (3.35 × 10^6^, *n* = 2).

Esters showed the most pronounced between-variety differences. Ethyl palmitate was significantly higher in SFGR (75.89 ± 19.30 × 10^6^) compared to SFLPC (16.80 ± 7.21 × 10^6^), representing a 4.5-fold difference (*p* = 0.008). Ethyl oleate showed a consistent directional trend toward higher absolute peak areas in SFGR (22.86 ± 12.11 × 10^6^) vs. SFLPC (6.50 ± 2.56 × 10^6^), with a 3.5-fold difference in mean values (*p* = 0.084, trend). Ethyl dodecanoate was detected exclusively in SFGR with high inter-replicate variability (44.87 ± 20.60 × 10^6^). Methyl palmitate showed no significant difference (SFLPC: 9.73 ± 5.70; SFGR: 9.23 ± 1.99 × 10^6^, ns). Ethyl oleate possesses documented anti-inflammatory properties [14], and the exclusive presence of 2,4-di-tert-butylphenol in SFGR—a phenolic compound with antifungal and antioxidant activities [15]—further distinguishes the glutinous rice fermentation profile.

The volatile profiles revealed qualitatively and quantitatively distinct characteristics between the two fermented rice varieties. SFGR was characterized by significantly higher long-chain fatty acid ethyl ester content, exclusive ethyl dodecanoate, exclusive 2,4-di-tert-butylphenol, and higher isobutyl alcohol, collectively contributing complex waxy–fatty notes with potential functional properties. SFLPC was distinguished by exclusive ethyl acetate formation. These differences reflect the combined influence of rice starch composition (high-amylose vs. waxy), substrate availability, and associated microbial metabolic pathways, confirming that rice variety selection significantly shapes the volatile profile of fermented rice products.

**Table 2:** Tentative annotation and absolute peak area of volatile compounds detected in sweet fermented Lueang Patew Chumphon rice (SFLPC) and sweet fermented glutinous rice (SFGR), based on mass spectral library matching and Linear Retention Index (LRI) comparison.

RT (min)	Compound Name	CAS No.	Class	LRI ^a^ (Calc.)	LRI ^b^ (Lit.)	ΔLRI	MS ^c^	SFLPC (×10^6^) ^d^	SFGR (×10^6^) ^d^	Ann. Level ^e^
1.6476	Acetaldehyde	75-07-0	Aldehyde	756	710	+46	>90	6.98 ± 0.88 ^a^	8.24 ± 1.14 ^a^	L1
4.7481	Isobutyl alcohol	78-83-1	Alcohol	1088	1090	−2	>90	23.53 ± 1.71 ^b^	33.09 ± 3.56 ^a^	L1
7.0767	Isoamyl alcohol	123-51-3	Alcohol	1194	1210	−16	>90	273.91 ± 22.65 ^a^	267.54 ± 28.78 ^a^	L1
14.2416	2-Ethyl-1-hexanol	104-76-7	Alcohol	1485	1490	−5	>90	n.d.	17.09 ± 2.51 †	L2
17.4846	Menthol	1195-79-5	Alcohol	1643	1640	+3	>90	16.55 ± 8.57 ^a^	24.80 ± 7.57 ^a^	L2
2.3091	Ethyl acetate	141-78-6	Ester	826	880	−54	>90	3.35 ± 0.00 ‡	n.d.	L1
21.0230	Ethyl dodecanoate	106-33-2	Ester	1984	1990	−6	>90	n.d.	44.87 ± 20.60	L2
25.8299	Methyl palmitate	112-39-0	Ester	2180	2185	−5	>90	9.73 ± 5.70 ^a^	9.23 ± 1.99 ^a^	L1
26.2796	Ethyl palmitate	628-97-7	Ester	2201	2208	−7	>90	16.80 ± 7.21 ^a^	75.89 ± 19.30 ^a^	L1
28.6859	Ethyl oleate	111-62-6	Ester	2372	2370	+2	>90	6.50 ± 2.56	22.86 ± 12.11	L1
8.9222	Acetoin	513-86-0	Ketone	1281	1285	−4	>90	20.75 ± 7.51 ^a^	21.26 ± 1.06 ^a^	L1
26.5541	2,4-Di-tert-butylphenol	96-76-4	Phenol	2213	2210	+3	>90	n.d.	detected	L2

^a^ Linear Retention Index calculated from n-alkane standards (C_7_–C_30_) under identical GC-MS conditions (Kovåts equation). ^b^ Literature LRI values from NIST Chemistry WebBook, and Flavnet database; polar stationary phase values. ^c^ MS: Match score vs. NIST 14/17 and Wiley 10 libraries. ^d^ Values represent mean absolute peak area (×10^6^) ± standard deviation (*n* = 3). Different superscript letters (^a^,^b^) within the same row indicate significant differences between SFLPC and SFGR (independent samples *t*-test, *p* < 0.05, after confirming variance homogeneity with Levene’s test). n.d. = not detected. † *n* = 2 valid replicates. ‡ *n* = 2 valid replicates (Rep 3 not detected). ^e^ Annotation confidence level: L1 = confirmed by retention time and LRI matching with authentic commercial reference standard under identical GC-MS conditions (MSI Level 1); L2 = tentative annotation based on MS library matching (>90%) and LRI comparison only (MSI Level 2). ΔLRI: Difference between calculated and literature LRI. Values within ±50 units indicate reliable annotation on polar columns [16,17]. GC-MS conditions: Agilent 7890B GC-7000D MS, VF-WAXms column (30 m × 0.25 mm × 0.25 µm); 40 °C (1 min) → 110 °C at 5 °C/min → 250 °C at 8 °C/min (5 min hold); helium carrier gas 1 mL/min.

### 2.3. Nutritional Characteristics

Mineral analysis revealed large effect sizes between varieties for key macrominerals (Table 3). SFLPC demonstrated large effect sizes for phosphorus (2.1-fold higher: 1445 ± 143 vs. 687 ± 54 mg/kg; Cohen’s d = 3.2, very large effect), potassium (1.9-fold higher: 1138 ± 0.3 vs. 612 ± 38 mg/kg; Cohen’s d = 2.8, very large effect), and magnesium (1.9-fold higher: 217 ± 15 vs. 115 ± 6 mg/kg; Cohen’s d = 2.1, large effect) compared to SFGR. Calcium showed moderate effect sizes with 53% higher levels in SFLPC (192 ± 2 vs. 125 ± 5 mg/kg). Among trace elements, zinc concentrations showed small effect sizes between varieties (9% difference: 19.7 ± 1.8 vs. 18.1 ± 0.8 mg/kg), while iron and copper demonstrated negligible effect sizes between varieties.

Fermentation produced moderate to large effect sizes in mineral composition compared to unfermented counterparts. Phosphorus showed moderate effect sizes with 37% increase in SFLPC and 23% increase in SFGR. Zinc demonstrated small to moderate effect sizes with 29% increase in SFLPC and 18% increase in SFGR. Sulfur content showed large effect sizes with substantial decreases of 70% in SFLPC and 66% in SFGR following fermentation.

Nutritional analysis revealed moderate effect sizes between varieties (Table 4). SFGR showed moderate effect sizes for carbohydrate content (4.6% higher than SFLPC: 85.14 ± 0.20% vs. 81.42 ± 0.12%), while SFLPC demonstrated moderate effect sizes for protein content (16% higher: 10.53 ± 0.07% vs. 9.07 ± 0.03%) and fat content (41% higher: 1.58 ± 0.06% vs. 1.12 ± 0.05%). Energy values showed small effect sizes with limited practical significance (1.3% difference: 386.9 ± 1.2 vs. 382.1 ± 0.7 kcal/100 g).

Fermentation produced large effect sizes in protein content compared to unfermented counterparts: 41% increase in SFGR and moderate effect sizes with 21% increase in SFLPC. The fermentation process showed large effect sizes in reducing crude dietary fiber content in both varieties, with substantial decreases likely due to enzymatic degradation during fermentation.

### 2.4. Quality Assessment and Bioactivity

Color measurement using the Lab* system [18] showed minimal differences between products (Table 5). Both fermented products met Thai Community Product Standards [19] for traditional sweet fermented rice, with acceptable visual characteristics for consumer acceptance.

Both fermented products demonstrated acceptable microbiological quality with no detectable *Escherichia coli* and yeast/mold counts below 100 CFU/g. The combination of pH reduction (≤3.55) and organic acid production (>6000 mg/L lactic acid) provided effective preservation against pathogenic microorganisms.

**Figure 1:**
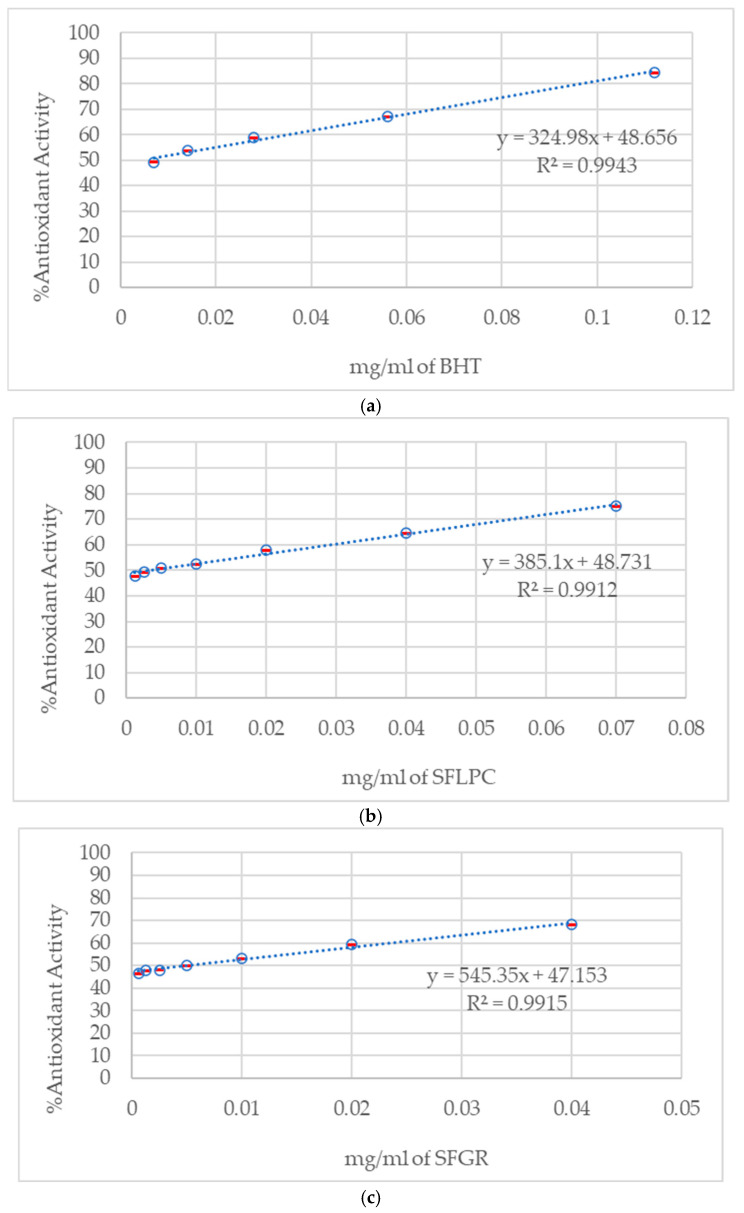
Antioxidant activity determination by DPPH radical scavenging assay showing dose–response relationships for (**a**) BHT standard control, (**b**) SFLPC (sweet fermented Lueang Patew Chumphon rice), and (**c**) SFGR (sweet fermented glutinous rice). Data points represent means ± standard deviation (*n* = 3). Linear regression parameters and correlation coefficients (R^2^) demonstrate excellent model fit for all treatments. Error bars indicate standard deviation and may appear small due to high analytical precision.

DPPH radical scavenging assay revealed statistically significant differences in antioxidant capacity between varieties (Figure 1). SFLPC demonstrated lower IC_50_ values (3.30 ± 0.15 μg/mL, *n* = 9) compared to SFGR (5.20 ± 0.25 μg/mL, *n* = 9), representing a 36% improvement in radical scavenging efficiency (*p* < 0.001, Student’s *t*-test). ANCOVA analysis confirmed significant treatment effect (F_1_,_16_ = 47.3, *p* < 0.001) with concentration as covariate. Both fermented products demonstrated antioxidant activity comparable to synthetic antioxidant BHT (IC_50_: 4.10 ± 0.20 μg/mL), suggesting potential applications as natural antioxidant sources.

Note on Figure 1: The dose–response curves show mean values (*n* = 3) with standard deviation error bars. Due to the high precision of the analytical method and small standard deviations (typically <5% of mean values), error bars may appear minimal but are present throughout the concentration range tested.

## 3. Discussion

### 3.1. Microbial Community Dynamics and Fermentation Processes

The microbial succession observed during sweet fermented rice production demonstrated complex interactions characteristic of traditional Asian fermented foods [20]. The initial presence of *Aspergillus* sp. and *Rhizopus stolonifer*, followed by their disappearance and emergence of *Candida tropicalis* alongside sustained *Pediococcus pentosaceus* populations, reflected sequential metabolic activities essential for fermentation optimization. This succession pattern aligns with established fermentation theory where fungi initiate starch hydrolysis, followed by yeast and lactic acid bacteria activities [20].

The enzymatic contributions of each microbial group facilitated distinct biochemical transformations. *Aspergillus* sp. and *R. stolonifer* produced α-amylase, glucoamylase, and proteases during initial phases, converting complex carbohydrates into fermentable substrates. *C. tropicalis* subsequently metabolized these sugars into ethanol and contributed to ester synthesis through alcohol acetyltransferase activity. *P. pentosaceus* maintained consistent lactic acid production throughout the process, establishing the preservation system through pH reduction [20,21].

The large effect size pH reductions observed (43% in SFLPC and 39% in SFGR) created environmental conditions that sequentially favored different microbial groups. The convergent final pH values (≤3.55) despite moderate effect size initial differences (0.37 units) demonstrated the robust control mechanisms inherent in this traditional fermentation system. This pH range effectively inhibited pathogenic microorganisms while maintaining beneficial fermentation microflora [20].

### 3.2. Carbohydrate Metabolism and Metabolic Constraints

The large effect size TSS increases (6.4-fold for SFLPC and 4.3-fold for SFGR) reflected extensive enzymatic starch hydrolysis during fermentation [22]. However, the substantial TSS difference between varieties (56% higher in SFGR with large effect size) did not translate to proportional ethanol production differences, which showed only small effect sizes (3% difference between varieties). This apparent paradox highlighted multiple metabolic constraints operating within the fermentation system [23].

The primary limitation appeared to be ethanol tolerance of *C. tropicalis* under specific fermentation conditions. Most yeast strains exhibit decreased fermentation efficiency as ethanol concentrations approach 3–4% *v*/*v*, particularly in high-acid environments. The toxic effects of ethanol on yeast cell membranes create natural ceilings for alcohol production, independent of substrate availability [24]. Additionally, the large effect size TSS concentration in SFGR (33.47 °Brix) may have induced osmotic stress, paradoxically inhibiting yeast metabolism [25]. High sugar levels can reduce cell viability and fermentation rates through osmotic pressure effects [22,26]. The dual fermentation system involving both *C. tropicalis* and *P. pentosaceus* also created substrate competition, where lactic acid bacteria simultaneously utilized glucose for organic acid production, limiting sugar availability for alcoholic fermentation [27,28].

### 3.3. Chemical Composition and Variety-Specific Characteristics

The volatile compound profiles between SFLPC and SFGR demonstrate clear rice variety-specific influences on fermentation-derived metabolites, consistent with previous studies on varietal effects in cereal fermentation [29]. Absolute peak area analysis showed that isoamyl alcohol—the dominant volatile via the Ehrlich pathway from leucine—was present at comparable levels in both varieties (273.91 vs. 267.54 × 10^6^, ns), suggesting similar overall capacity for amino acid catabolism across both fermentation systems [10]. In contrast, isobutyl alcohol (derived from valine via the Ehrlich pathway) was significantly higher in SFGR (*p* = 0.014), suggesting that valine availability or catabolism may differ between the rice varieties. The 4.5-fold higher ethyl palmitate in SFGR (*p* = 0.008) and the consistent trend toward higher ethyl oleate (3.5-fold, *p* = 0.084) are consistent with greater fatty acid precursor availability in high-amylopectin waxy rice, supporting enhanced ester synthase activity during glutinous rice fermentation [30]. Exclusive ethyl dodecanoate (44.87 ± 20.60 × 10^6^) further reinforces this lipid-mediated esterification pattern in SFGR [31]. The exclusive presence of ethyl acetate in SFLPC, characteristic of early-stage ester synthesis, distinguishes the high-amylose fermentation profile.

The observed volatile differences stem from fundamental compositional distinctions: high-amylose LPC rice (22–25% amylose) versus waxy glutinous rice (>95% amylopectin) present structurally different fermentation substrates affecting microbial metabolism [32]. The degree of gelatinization analysis confirmed both cooking methods achieved adequate starch gelatinization (32.5–40.1%), validating that volatile differences arise from intrinsic rice variety characteristics rather than preparation variations. Acetoin levels (1.73–2.06%) and low acetaldehyde (0.66–0.71%) indicate balanced, healthy fermentation across both varieties [33].

These variety-specific profiles demonstrate that rice selection critically determines both sensory and functional attributes of fermented products. SFLPC offers traditional alcoholic-fruity aromatics valued in Southeast Asian fermented foods, while SFGR provides complex waxy–fatty notes complemented by bioactive compounds potentially enhancing product stability and health benefits. The successful fermentation of high-amylose LPC rice expands utilization of non-traditional varieties in fermentation applications, supporting agricultural diversification and value-added product development. These findings enable producers to develop differentiated products tailored to specific consumer preferences or functional applications, contributing to both market diversification and sustainable utilization of diverse rice genetic resources.

Mineral composition analysis revealed large effect sizes for key macrominerals, with SFLPC demonstrating 2.1-fold higher phosphorus, 1.9-fold higher potassium, and 1.9-fold higher magnesium concentrations compared to SFGR. These differences likely reflect both inherent rice variety characteristics and fermentation-induced modifications. The moderate to large effect sizes observed in mineral enhancement following fermentation (37% phosphorus increase in SFLPC, 29% zinc increase) suggest improved bioavailability through microbial enzyme activity, potentially breaking down phytic acid and other mineral-binding compounds [34].

Proximate composition revealed moderate effect sizes between varieties, with SFGR showing 4.6% higher carbohydrate content while SFLPC demonstrated 16% higher protein and 41% higher fat content. The large effect sizes in protein enhancement following fermentation (41% increase in SFGR, 21% increase in SFLPC) indicated significant nutritional improvements compared to unfermented counterparts.

### 3.4. Quality Assessment and Bioactive Properties

The moderate effect size differences in antioxidant capacity (36% better scavenging in SFLPC) demonstrated variety-specific bioactive compound development during fermentation. The IC_50_ values (3.3 µg/mL for SFLPC vs. 5.2 µg/mL for SFGR) represented practically meaningful differences in radical scavenging ability, with SFLPC outperforming synthetic antioxidant BHT by 20%.

Color characteristics showed small to moderate effect sizes between varieties, with measurable differences in redness (29% difference) and yellowness (10% difference) that may influence consumer acceptance. Despite these differences, both products met established quality standards, demonstrating the robustness of the traditional fermentation process across different rice varieties.

The microbiological safety evaluation confirmed that the dual preservation system of pH reduction and organic acid production effectively controlled pathogenic microorganisms while maintaining product quality [35]. The absence of detectable *E. coli* and acceptable yeast/mold counts validated the safety profile of both fermented products.

## 4. Materials and Methods

### 4.1. Materials

#### 4.1.1. Starter Cakes

Traditional fermentation starter cakes (Look Pang) used for Thai sweet fermented rice production were obtained from a single local producer in Muang District, Chumphon Province, Thailand, to maintain consistency throughout the study. These semicircular starch-based inoculants contain indigenous molds and yeasts specific to regional production methods. The starter cakes are produced according to traditional Thai fermentation standards and are supplemented with locally available herbs primarily to inhibit spoilage microorganisms and maintain product quality rather than to introduce specific microbial strains. The starter cakes measured 3.5 cm in diameter with an average weight of 12.02 g, and were stored at 4 °C until use.

To ensure reproducibility, all starter cakes used in this study were sourced from the same producer and production batch. The consistency of the starter cake performance was verified through preliminary trials conducted over a 2-year period, which demonstrated stable fermentation parameters including pH, total soluble solids (TSS), and alcohol content, as well as consistent microbial community profiles typical of traditional Thai sweet rice fermentation.

#### 4.1.2. Rice Varieties

Lueang Patew Chumphon (LPC) rice, a local variety, was procured from Bang Son Subdistrict Community Enterprise, Chumphon Province, Thailand. Commercial glutinous rice was purchased from a local supermarket and served as a comparative control.

#### 4.1.3. Chemicals and Reagents

Potato dextrose agar (PDA) and nutrient agar were purchased from HIMEDIA (Mumbai, India). All other chemicals and reagents used were of analytical grade unless otherwise specified.

### 4.2. Methods

#### 4.2.1. Microbial Identification of Starter Cake Isolates

A total of 1 g of finely ground starter cake was suspended in 9 mL sterile distilled water, vortexed, and subjected to 10-fold serial dilution. Aliquots (0.5 mL) of each dilution were spread-plated on PDA and incubated at 28 °C for 5 days. Fungal isolates were purified using the streak plate method until pure cultures were obtained.

Molecular identification was performed using DNA sequencing of the internal transcribed spacer (ITS) region for eukaryotic organisms and 16S rRNA gene for prokaryotic organisms. DNA extraction utilized the DNeasy Plant Mini Kit (Qiagen, Dusseldorf, Germany) according to manufacturer instructions. PCR amplification employed ITS1/ITS4 primers for fungi and 27f/1492r primers for bacteria with high-fidelity Taq polymerase.

Amplified products were purified using QIAquick PCR Purification Kit (Qiagen) and submitted for Sanger sequencing (Macrogen Inc., Seoul, Republic of Korea). Sequence quality assessment included chromatogram evaluation and trimming of low-quality terminal regions. Extracted DNA sequences were analyzed using BLAST (Basic Local Alignment Search Tool, https://blast.ncbi.nlm.nih.gov/Blast.cgi, accessed on 20 January 2026) against the NCBI nucleotide database using standard parameters for highly similar sequences (megablast algorithm).

Species identification criteria required sequence similarity ≥99% compared to type strain sequences. Multiple independent BLAST searches confirmed consistency of identification results. The NCBI BLAST database provides continuously updated reference sequences from validated taxonomic sources, ensuring reliable species-level identification without requiring version-specific database citations.

Cross-validation using MALDI Biotyper (Bruker Daltonik GmbH, Germany) provided supplementary identification confirmation for culturable isolates, with score values ≥2.0 indicating reliable species identification.

#### 4.2.2. Sweet Fermented Rice Preparation

Glutinous rice fermentation: Glutinous rice (500 g) was washed with tap water and soaked in filtered water for 6 h, then steamed for 45 min at atmospheric pressure (100 °C). After cooling to ambient temperature (28 ± 2 °C), the rice was rinsed with filtered water to remove excess surface starch. The cooked rice was spread on sterile trays in a laminar flow hood for 15 min to achieve appropriate moisture content (65–70%). Pulverized starter cake (6 g, representing 1.2% *w*/*w* inoculum) was thoroughly mixed with the cooled rice under aseptic conditions using sterilized utensils. The inoculated rice was transferred to sterile airtight glass bottles (500 mL capacity). Fermentation was conducted at 32 ± 1 °C and 75 ± 5% relative humidity for 48 h in a controlled incubation chamber.

LPC rice fermentation: LPC rice (500 g) was washed three times with filtered water and cooked using an electric rice cooker (Panasonic SR-DF101, Osaka, Japan) with a rice-to-water ratio of 1:1.2 (*w*/*v*) to achieve complete gelatinization. Following identical cooling and mixing procedures as described above, the cooked LPC rice was inoculated with 6 g of starter cake powder and incubated under identical controlled conditions (32 ± 1 °C, 75 ± 5% RH, 48 h).

##### Rationale for Variety-Specific Cooking Methods

The selection of cooking methods was based on fundamental differences in starch composition between the rice varieties. Glutinous rice contains >95% amylopectin with minimal amylose [36], while LPC rice contains approximately 22–25% amylose [5]. These compositional differences necessitate distinct cooking approaches to achieve optimal gelatinization while maintaining structural integrity suitable for fermentation. Steaming is the traditional and scientifically optimal method for glutinous rice, preventing grain disintegration that occurs with absorption cooking in high-amylopectin rice [37]. Conversely, the high amylose content of LPC rice requires absorption cooking (rice-to-water ratio 1:1.2 *w*/*v*) to achieve complete gelatinization without excessive starch leaching or grain breakdown [38]. These methods reflect authentic traditional Thai fermented rice (khao mak) preparation practices.

#### 4.2.3. Degree of Gelatinization Determination

To validate that both cooking methods achieved comparable starch gelatinization despite different procedures, the degree of gelatinization (DG) of cooked rice samples was determined using the amylose-iodine complex formation method according to Baks et al. [39] with minor modifications. This dual-treatment approach differentiates between gelatinized and total (gelatinized plus native) starch fractions.

For gelatinized starch determination (A_1_), cooked rice flour (20 mg, dried and ground to pass through 80-mesh sieve) was dissolved in 0.15 M KOH solution (25 mL) and stirred at room temperature for 15 min. The suspension was centrifuged at 5000 rpm (2880× *g*) for 5 min. An aliquot of the supernatant (1 mL) was mixed with 0.17 M HCl (9 mL) and iodine solution (1 mL, 2.5% I_2_/KI solution prepared by dissolving 2.5 g I_2_ and 25 g KI in 100 mL distilled water). After thorough mixing, absorbance was measured at 600 nm using a UV-visible spectrophotometer (UV-1800, Shimadzu, Kyoto, Japan) against a reagent blank.

For total starch determination (A_2_), cooked rice flour (20 mg) was dissolved in 0.40 M KOH solution (25 mL) and heated at 95 °C for 10 min in a water bath to ensure complete starch dispersion. After cooling to room temperature, the solution was centrifuged at 5000 rpm (2880× *g*) for 5 min. The aliquot of the supernatant (1 mL) was mixed with 0.45 M HCl (9 mL) and iodine solution (1 mL, 2.5%). After mixing, absorbance was measured at 600 nm against a reagent blank. The degree of gelatinization (%) was calculated using the equation:

DG (%) = (A_1_/A_2_) × 100

where A_1_ represents the absorbance of gelatinized starch and A_2_ represents the absorbance of total starch. All measurements were performed in triplicate, and results are expressed as mean ± standard deviation.

#### 4.2.4. Chemical Analyses

##### pH Measurement

The pH of fermented rice samples was homogenized with deionized water (1:1 *w*/*v*), vortexed for 30 s, and centrifuged at 4800 rpm for 10 min. The supernatant was filtered through membrane filtration before pH measurement using a C1010 multi-parameter analyzer (Consort bvba, Turnhout, Belgium). All measurements were performed in triplicate.

##### Total Soluble Solids Determination

Total soluble solid content (°Brix) was measured in extracted juice using an Atago PAL-1 (3810) Digital Hand-held Pocket Refractometer (Tokyo, Japan).

##### Ethanol Quantification

Ethanol content was determined using Headspace—Gas Chromatography with Flame Ionization Detection (HS-GC/FID) on an Agilent 6850 system (Agilent Technologies, Santa Clara, CA, USA). Headspace parameters: sample size: 1 g in 20 mL headspace vials with the incubation temperature: 80 °C for 30 min; mixing: 500 rpm during incubation. Separation was achieved using an HP INNOWAX column [30 m × 0.32 mm × 0.25 μm film thickness]. Sample injection (1 μL) was performed in split mode (50:1 ratio). Operating conditions included helium carrier gas (10 mL/min), hydrogen (30 mL/min), air (300 mL/min), injector temperature (180 °C), and detector temperature (250 °C). The oven temperature program started at 50 °C (9 min hold) and ramped to 200 °C at 5 °C/min, with a final hold for 2 min. Total run time was 39 min. Ethanol identification confirmed by retention time matching with authentic standard and co-injection verification.

##### Lactic Acid Determination

Lactic acid content was analyzed using High-Performance Liquid Chromatography (HPLC) on an Agilent 1200 series system (Agilent Technologies, Santa Clara, CA, USA) equipped with a diode array detector (210 nm detection wavelength). Separation was performed using a LiChrospher 100 RP-18 column (250 × 4.0 mm, 5 μm particle size). The mobile phase consisted of 0.1% phosphoric acid in water at a flow rate of 1.0 mL/min. Injection volume was 20 μL, with column temperature maintained at 25 °C, and the run time was 20 min.

Sample preparation involved centrifugation of the fermented product liquid at 4800 rpm for 10 min, filtration through 0.22 μm membrane filters, and 50-fold dilution with deionized water. Sodium L-lactate (98% purity, Sigma-Aldrich, St. Louis, MO, USA) served as the analytical standard. The limit of quantification (LOQ) was 5 mg/L. Concentrations were determined using peak area integration and external standard calibration.

##### Volatile Compound Analysis

Volatile compounds were identified using Gas Chromatography—Tandem Mass Spectrometry (GC-MS/MS) on an Agilent 7890B GC-7000D MS system via Headspace—Solid Phase Microextraction (HS-SPME-GC-EI/MS). A Carboxen/DVB/PDMS fiber (2 cm, 50/30 μm, StableFlex™, Agilent Technologies) was used for extraction.

Optimized extraction conditions included the following: incubation temperature: 80 °C which was selected to ensure the effective extraction of high-molecular-weight compounds, such as ethyl palmitate and ethyl oleate; incubation time: 30 min; extraction time: 10 min; and desorption time: 2 min. Sample aliquots (1 mL) were placed in 20 mL SPME vials sealed with PTFE/silicone septa.

Chromatographic separation utilized an Agilent CP9205 VF-WAXms capillary column (30 m × 0.25 mm × 0.25 μm) with helium carrier gas (1 mL/min constant flow, 7.0699 psi). Instrument parameters: electron ionization: 70 eV; splitless injection at 250 °C, oven temperature program from 40 °C (1 min) to 110 °C at 5 °C/min, then to 250 °C at 8 °C/min with a 5-min final hold. Volatile compounds were identified by mass spectral matching (NIST 14/17, Wiley 10) with match factors >90%, and further confirmed by calculating Linear Retention Index (LRI) values [39,40] using n-alkane standards (C_7_–C_30_) run under identical chromatographic conditions. Volatile compound data are reported as mean absolute peak area (×10^6^ ± SD, *n* = 3). In HS-SPME/GC-MS comparative analyses, absolute peak areas obtained under strictly controlled and identical extraction and instrumental conditions are directly comparable between samples and represent the appropriate reporting unit, avoiding the compositional bias introduced by normalization to a total peak area sum [12,13].

##### Mineral Content Analysis

Fermented rice samples were oven-dried at 70 °C and ground to pass through a 40-mesh sieve. Nitrogen content was determined by Kjeldahl digestion, distillation, and titration. Other mineral elements (P, S, K, Ca, Mg, Fe, Cu, Zn, Na) were analyzed following Momen et al. [40].

Samples were incinerated in a muffle furnace (Carbolite AAF 1100, Carbolite, Hope, UK) at 550 °C for 5 h. Ash samples were dissolved in 10 mL of 1 N HCl and analyzed using Inductively Coupled Plasma—Optical Emission Spectrometry (ICP-OES, Perkin Elmer AVIO 500, Shelton, CT, USA).

##### Proximate Composition Analysis

Proximate composition, including moisture, ash, protein, fat, and dietary fiber, was determined using standard AOAC methods. Moisture content was measured using a hot air circulating oven at 70 °C. Ash content was determined gravimetrically by incineration at 550 ± 5 °C (AOAC Method 930.05) [41]. Total protein content was determined by the Kjeldahl method (AOAC Method 978.04) using a nitrogen conversion factor of 5.95 on a Kjeltec 8400 analyzer (FOSS, Hillerød, Denmark) [41]. Fat content was determined by acid hydrolysis (4N HCl) followed by petroleum ether extraction (AOAC Method 930.09) using a SoxTec 8000 system (FOSS, Denmark) [41]. Dietary fiber was determined according to AOAC Method 930.10 [42]. Total carbohydrate content was calculated by difference, as follows:

%Total Carbohydrate = 100 − (%Protein + %Fat + %Moisture + %Ash + %Dietary Fiber)(1)

##### Caloric Value Determination

Food energy values (kcal/100 g) were calculated using Atwater conversion factors, namely, protein (4 kcal/g), carbohydrate (4 kcal/g), and fat (9 kcal/g):

Food energy value = (%protein × 4) + (%carbohydrate × 4) + (%fat × 9)(2)

#### 4.2.5. Physical Characterization

##### Color Measurement

Color parameters were measured using a Hunter Lab Colorimeter (Chroma Meter CR-400, Konica Minolta, Tokyo, Japan) and recorded as L*, a*, and b* values, where L* represents lightness (0–100 scale), a* represents green (−) to red (+) chromaticity, and b* represents blue (−) to yellow (+) chromaticity.

#### 4.2.6. Microbiological Analyses

##### Microbial Identification of Final Products

Pure cultures were isolated from fermented products using the cross-streak method on nutrient agar plates. Microorganisms were identified using MALDI-TOF MS (MALDI Biotyper, Bruker Daltonik GmbH, Bremen, Germany).

##### Microbiological Quality Assessment

Microbiological quality was evaluated according to the Thai Community Product Standard (TCPS 162/2003) for Khao Mak [17]. *Escherichia coli*, total coliforms, yeasts, and molds were enumerated using 3M™ Petrifilm™ *E. coli*/Coliform Count Plates and Rapid Yeast and Mold Count Plates (3M Company, St. Paul, MN, USA), respectively.

A total of 25 g of sample was homogenized with 225 mL sterile buffered peptone water and subjected to 10-fold serial dilutions. One-milliliter aliquots at dilutions of 10^−1^, 10^−2^, and 10^−3^ were plated on Petrifilm and incubated at 37 °C for 24 h (*E. coli*) and 28 °C for 3–5 days (yeast and mold). Results were expressed as colony-forming units per gram (CFU/g).

#### 4.2.7. Antioxidant Activity Determination

Free radical scavenging activity was assessed using the DPPH assay according to Seethalaxmi et al. [42] with modifications for enhanced statistical rigor. Sample preparation involved fermented rice homogenization (1:5 *w*/*v* with distilled water), centrifugation (4800 rpm, 10 min), and filtration through 0.22 μm membranes. Six serial dilutions were prepared for each sample to establish complete dose–response curves. Sample aliquots (2 mL) at concentrations ranging from 0.5 to 20 μg/mL were mixed with 5 mL of 0.1 mM methanolic DPPH solution, vortexed for 30 s, and incubated in darkness at room temperature for 30 min. Absorbance measurements at 517 nm were made using a UV-visible spectrophotometer (Thermo Fisher Scientific, Waltham, MA, USA) with methanol as blank.

Statistical analysis employed nonlinear regression (four-parameter logistic model) to determine IC_50_ values with 95% confidence intervals. Between-sample comparisons were analyzed using independent samples *t*-test. All determinations were performed in triplicate with independent sample preparations.

Free radical scavenging activity was calculated as follows:

Scavenging activity (%) = [(A_0_ − (A_1_ − A_2_))/A_0_] × 100 (3)

where

A_0_ = absorbance of control (DPPH only);

A_1_ = absorbance of sample + DPPH;

A_2_ = absorbance of sample blank.

IC_50_ values representing 50% inhibition were determined by plotting inhibition percentage against concentration. All measurements were performed in triplicate.

#### 4.2.8. Statistical Analysis

All experiments were conducted in triplicate, with the results expressed as means ± standard deviation (SD). Statistical analyses were performed using SPSS software (version 23.0, SPSS Inc., Chicago, IL, USA). Data normality was assessed using the Shapiro–Wilk test.

Temporal changes within each rice variety were analyzed using repeated measures ANOVA followed by Bonferroni post hoc comparisons. The sphericity assumption was evaluated using Mauchly’s test, with Greenhouse-Geisser correction applied when violated. Between-variety comparisons (SFLPC vs. SFGR) were analyzed using an independent samples *t*-test after confirming data normality with the Shapiro–Wilk test and variance homogeneity with Levene’s test. Statistical significance was set at *p* ≤ 0.05.

## 5. Conclusions

This study demonstrates that high-amylose Lueang Patew Chumphon (LPC) rice represents a viable and advantageous substrate for traditional sweet fermented rice production. Successful fermentation was validated through consistent pH reduction (43% to 3.5), lactic acid production (>6 g/L), and appropriate alcohol formation (3.6% *v*/*v*), confirming the adaptability of traditional Thai fermentation systems to non-glutinous rice varieties. The degree of gelatinization analysis confirmed that variety-specific cooking methods achieved comparable starch gelatinization (32.5–40.1%), indicating that observed differences arose from intrinsic rice characteristics rather than processing variations.

GC-MS analysis with mass spectral library matching and LRI comparison tentatively annotated twelve volatile compounds, with eight confirmed at MSI Level 1 using authentic reference standards. Absolute peak area analysis revealed distinct variety-specific volatile profiles: SFGR exhibited significantly higher absolute peak areas of ethyl palmitate (4.5-fold, *p* = 0.008) and isobutyl alcohol (40%, *p* = 0.014), along with exclusive ethyl dodecanoate and 2,4-di-tert-butylphenol, contributing complex waxy–fatty notes with potential bioactive properties. SFLPC was characterized by exclusive ethyl acetate production and comparable isoamyl alcohol content to SFGR. These variety-specific volatile profiles, arising from intrinsic rice compositional differences validated by degree of gelatinization analysis, demonstrate that rice variety selection critically determines the volatile characteristics of fermented rice products. The microbial succession pattern—from fungal starch hydrolysis through yeast fermentation to sustained lactic acid production—remained consistent across both rice varieties, demonstrating system robustness. SFLPC demonstrated significant nutritional advantages, including 2.1-fold higher phosphorus (1445 vs. 687 mg/kg), 1.9-fold higher potassium and magnesium, 16% higher protein content, and 36% superior antioxidant capacity (IC_50_: 3.30 vs. 5.20 μg/mL), surpassing synthetic antioxidant BHT by 20%. These enhancements, combined with maintained microbiological safety, establish SFLPC as a nutritionally superior fermented product with strong potential for functional food applications. Furthermore, rice variety selection was found to influence multiple aspects of fermented product quality through large to moderate effect sizes across chemical, nutritional, and sensory parameters, underscoring the importance of substrate selection in product differentiation.

The variety-specific metabolic pathways and compound development patterns identified in this study suggest opportunities for targeted optimization in commercial applications. The maintenance of traditional fermentation benefits combined with variety-specific enhancements represents a sustainable approach to functional food development that simultaneously preserves cultural food heritage and supports agricultural diversification through the value-added utilization of diverse rice genetic resources.

These findings address critical knowledge gaps in high-amylose rice utilization and provide scientific validation for traditional fermentation practices. Future research should investigate sensory evaluation with trained consumer panels, fermentation optimization for high-amylose substrates, glycemic response assessment, and scale-up feasibility for commercial production. This research establishes high-amylose LPC rice as a promising substrate for traditional fermentation, offering enhanced nutritional properties and unique aromatic characteristics while preserving traditional fermentation knowledge.

## Figures and Tables

**Table 1:** Physicochemical changes during fermentation of sweet fermented rice prepared from different rice varieties.

Parameter	Sweet Fermented Lueang Patew Chumphon Rice (SFLPC)			Sweet Fermented Glutinous Rice (SFGR)		
	Day0	Day1	Day2	Day0	Day1	Day2
pH	6.15 ± 0.02	3.62 ± 0.01	3.50 ± 0.01	5.78 ± 0.11	3.65 ± 0.02	3.55 ± 0.00
TSS (°Brix)	3.33 ± 0.31	19.53 ± 0.15	21.47 ± 0.06	7.77 ± 0.40	32.03 ± 0.40	33.47 ± 0.12
Ethanol (%*v*/*v*)	0.03 ± 0.00	1.77 ± 0.04	3.60 ± 0.11	0.03 ± 0.00	1.87 ± 0.06	3.72 ± 0.12
Lactic acid (μg/L)	2.49 ± 11.82	5.89 ± 37.56	6.06 ± 17.77	2.48 ± 13.93	5.84 ± 9.49	6.04 ± 9.98

Each value represents mean ± standard deviation (*n* = 3).

**Table 3:** Mineral concentrations (mg/kg) in the final fermented products and their corresponding cooked rice controls.

Mineral	SFLPC	SFGR	LPC	GR
Calcium (Ca)	191.7 ± 1.6 ^b^	125.1 ± 4.6 ^c^	218.1 ± 14.7 ^a^	208.1 ± 9.8 ^ab^
Phosphorus (P)	1444 ± 143 ^a^	686.7 ± 54.2 ^c^	1055 ± 16 ^b^	556.5 ± 7.8 ^c^
Magnesium (Mg)	217.0 ± 14.7 ^a^	115.0 ± 6.1 ^c^	223.8 ± 5.2 ^a^	133.7 ± 2.3 ^b^
Sodium (Na)	98.76 ± 3.62 ^c^	85.01 ± 7.28 ^d^	199.1 ± 7.7 ^a^	176.0 ± 5.9 ^b^
Potassium (K)	1138 ± 0 ^b^	611.6 ± 38.2 ^d^	1216 ± 44 ^a^	1052 ± 23 ^c^
Sulphur (S)	7.20 ± 1.07 ^d^	15.24 ± 1.96 ^c^	23.75 ± 0.27 ^b^	44.61 ± 4.43 ^a^
Iron (Fe)	5.90 ± 0.08 ^c^	5.62 ± 0.52 ^c^	14.89 ± 0.32 ^a^	7.53 ± 0.44 ^b^
Zinc (Zn)	19.66 ± 1.80 ^a^	18.05 ± 0.75 ^a^	15.19 ± 0.19 ^b^	15.28 ± 0.42 ^b^
Copper (Cu)	2.84 ± 0.51 ^c^	2.70 ± 0.54 ^c^	7.20 ± 0.67 ^a^	5.63 ± 0.10 ^b^

Values with different superscript letters on the same row indicate a significant difference (*p* < 0.05). Each value represents the mean of three replicates with standard deviation. Abbreviations: SFLPC, sweet fermented Lueang Patew Chumphon rice; SFGR, sweet fermented glutinous rice; LPC and GR mean cooked rice from Lueang Patew Chumphon rice and steamed glutinous rice, respectively.

**Table 4:** Proximate composition and energy value of the final fermented products and their corresponding cooked rice controls.

Proximate Composition	SFLPC	SFGR	LPC	GR
%Carbohydrate	81.42 ± 0.12 ^b^	85.14 ± 0.20 ^a^	81.18 ± 0.06 ^b^	76.28 ± 0.47 ^c^
%Crude protein	10.53 ± 0.07 ^a^	9.07 ± 0.03 ^b^	8.73 ± 0.01 ^c^	6.43 ± 0.01 ^d^
%Crude fat	1.58 ± 0.06 ^a^	1.12 ± 0.05 ^c^	1.28 ± 0.02 ^b^	0.69 ± 0.04 ^d^
%Crude dietary fiber	2.61 ± 0.02 ^c^	1.61 ± 0.03 ^d^	5.47 ± 0.02 ^a^	4.33 ± 0.03 ^b^
%Ash	0.59 ± 0.03 ^a^	0.24 ± 0.02 ^c^	0.50 ± 0.03 ^b^	0.18 ± 0.02 ^d^
%Moisture content	3.27 ± 0.18 ^a^	2.81 ± 0.24 ^c^	2.84 ± 0.01 ^b^	2.10 ± 0.21 ^d^
Food energy value (kcal/g)	382.1 ± 0.7 ^b^	386.9 ± 1.2 ^a^	371.2 ± 0.2 ^c^	337.0 ± 0.8 ^d^

Values with different superscript letters on the same row indicate a significant difference (*p* < 0.05). Each value represents the mean of three replicates with standard deviation. Abbreviations: SFLPC, sweet fermented Lueang Patew Chumphon rice; SFGR, sweet fermented glutinous rice; LPC and GR mean cooked rice from Lueang Patew Chumphon rice and steamed glutinous rice, respectively.

**Table 5:** Color characteristics of the final fermented products.

Treatment	Color		
	L	a	b
SFLPC	79.77 ± 0.12 ^a^	−4.28 ± 0.00 ^a^	11.11 ± 0.02 ^b^
SFGR	78.51 ± 0.95 ^a^	−5.53 ± 0.33 ^b^	12.20 ± 0.07 ^a^

Values with different superscript letters in the same column indicate a significant difference (*p* < 0.05). Each value represents the mean of three replicates with standard deviation. Abbreviations: SFLPC, sweet fermented Lueang Patew Chumphon rice; SFGR, sweet fermented glutinous rice.

## Data Availability

Data is contained within the article.
